# Polyhydroxybutyrate (PHB): Critical Perspectives on Material Properties, Production Advances, and Challenges Toward Sustainable Commercialisation

**DOI:** 10.3390/ma19143013

**Published:** 2026-07-13

**Authors:** Veshara Ramdas, Sudhakar Muniyasamy, Sesethu Gift Njokweni, Parsons Letsoalo, Santosh Omrajah Ramchuran

**Affiliations:** 1 Bioprocessing Development Group, Chemicals Cluster, Council for Scientific and Industrial Research (CSIR), Meiring Naude Road, Brummeria, Pretoria 0184, South Africa; snjokweni@csir.co.za (S.G.N.); pletsoalo1@csir.co.za (P.L.); sramchuran@csir.co.za (S.O.R.); 2 Advanced Polymers and Composites Group, Chemicals Cluster, Council for Scientific and Industrial Research (CSIR), Meiring Naude Road, Brummeria, Pretoria 0184, South Africa

**Keywords:** polyhydroxyalkanoates (PHAs), bioplastics, microbial synthesis, biopolymer blends and composites, materials processing, waste-derived feedstocks, biodegradability, sustainable materials

## Abstract

Polyhydroxybutyrate (PHB), a microbial polyester belonging to the polyhydroxyalkanoate (PHA) family, has emerged as one of the most promising biodegradable alternatives to conventional petroleum-derived plastics. Its inherent marine biodegradability (typically mineralizing within months depending on material geometry and ambient temperature), biocompatibility, and ability to be synthesised from renewable and waste-derived feedstocks position PHB as a key candidate for supporting the transition towards a circular bioeconomy. Despite these advantages, widespread commercial adoption remains limited by high production costs, processing challenges, and performance constraints relative to established commodity plastics and competing biopolymers. This review critically evaluates the current state of PHB development from the perspective of sustainable commercialisation. Key aspects discussed include microbial biosynthesis pathways, feedstock selection, upstream fermentation strategies, downstream recovery technologies, and technoeconomic considerations influencing industrial feasibility. The intrinsic thermal, mechanical, and degradation characteristics of PHB are examined alongside modification approaches such as copolymerisation, polymer blending, plasticisation, and composite reinforcement that have been developed to overcome certain inherent physical–mechanical properties, narrow processing windows, and limited functional performance. Furthermore, characterisation methodologies, environmental degradation behaviour, and emerging industrial applications are assessed within the context of market requirements and sustainability objectives. Particular emphasis is placed on identifying the interconnected technical and economic bottlenecks that continue to hinder large-scale deployment, including feedstock costs, fermentation scalability, downstream processing expenses, and material performance trade-offs. By integrating advances across the entire PHB value chain, this review highlights current opportunities, remaining challenges, and future priorities required to enable the sustainable and economically viable commercialisation of PHB-based materials.

## 1. Introduction

The global reliance on conventional petroleum-based plastics has resulted in severe environmental consequences, including persistent pollution, microplastic accumulation, and increasing pressure on waste management systems. While these materials offer durability, versatility, and low production costs, their resistance to degradation has created long-term ecological challenges. In response, significant research efforts have been directed toward the development of biobased and biodegradable alternatives. However, the assumption that all bioplastics inherently offer sustainable solutions has increasingly been challenged, particularly when considering trade-offs between environmental performance, material functionality, and economic viability [1].

Among emerging alternatives, PHAs have attracted considerable attention due to their microbial origin and inherent biodegradability [2]. Within this family, PHB is the most extensively studied homopolymer, synthesised intracellularly by a wide range of microorganisms as an energy storage compound under nutrient-limited conditions. PHB exhibits several favourable characteristics, including biodegradability in diverse environments, biocompatibility, and the potential for production from renewable and waste-derived feedstocks [3]. These attributes position PHB as a promising candidate for sustainable material applications across packaging, biomedical, and agricultural sectors. Furthermore, the ability of PHB-producing microorganisms to utilise agricultural residues, industrial by-products, wastewater streams, and other waste-derived substrates aligns PHB with emerging circular bioeconomy and waste valorisation strategies. Nevertheless, feedstock selection remains closely linked to process performance, polymer quality, and overall production economics.

Despite these advantages, the translation of PHB from laboratory-scale research to large-scale commercial deployment remains limited. This gap highlights a critical disconnect between material potential and industrial feasibility. In practice, PHB faces several well-documented challenges, including high production costs relative to conventional plastics, energy- and solvent-intensive downstream processing, and inherent material limitations such as brittleness, low impact resistance, and a narrow thermal processing window [4,5]. These constraints are further compounded when PHB is evaluated against competing bioplastics, such as polylactic acid (PLA) and polybutylene adipate terephthalate (PBAT), which often offer more favourable processing characteristics or cost structures despite their own limitations [6,7,8,9].

Current research has made notable progress in addressing these challenges through strategies such as the utilisation of low-cost and waste-derived substrates, metabolic and genetic engineering of production strains, and material modification approaches including copolymerisation and polymer blending [10,11,12]. While these advancements have improved specific aspects of PHB performance and production efficiency, they have not yet fully resolved the fundamental bottlenecks that hinder its commercial competitiveness. In many cases, improvements in one domain introduce trade-offs in another; for example, enhanced flexibility through blending may increase formulation complexity, alter biodegradation behaviour, or increase costs [3,13].

A critical evaluation of these interdependencies is therefore necessary, particularly because the commercial feasibility of PHB is influenced not by any single limitation, but by the cumulative effects of feedstock selection, fermentation performance, downstream recovery efficiency, material properties, degradation behaviour, and market competitiveness. However, much of the existing literature remains fragmented, often focusing on isolated aspects of PHB development without integrating production, performance, environmental considerations, and economic feasibility within a unified framework. This lack of holistic assessment limits the ability to identify the key barriers preventing large-scale adoption.

In this context, the present review provides a critical and integrative assessment of PHB across its full value chain. The review is structured to examine (i) microbial production pathways and feedstock strategies, (ii) physicochemical and mechanical properties in comparison with conventional polymers and alternative bioplastics, and (iii) modification strategies aimed at improving material performance. These aspects are analysed through the lens of scalability, technoeconomic feasibility, lifecycle sustainability, and end-of-life environmental performance, with particular emphasis placed on identifying and synthesising the major bottlenecks to commercialisation. By bridging the gap between laboratory innovation and industrial implementation, this review aims to contribute not only to the scientific understanding of PHB but also to the development of actionable insights required to enable its transition into a commercially viable and sustainable material solution.

## 2. Biobased and Biodegradable Polymers: Conceptual Framework and Implications for PHAs

Bioplastics have emerged as promising alternatives to conventional petroleum-based polymers due to their potential to reduce dependence on fossil resources and mitigate environmental pollution. These materials are typically classified based on two independent criteria: their origin (biobased versus fossil-based) and their end-of-life behaviour (biodegradable versus non-biodegradable) [14]. While this classification provides a useful conceptual framework, it does not inherently reflect the overall sustainability or performance of a given material.

Biobased polymers or bioplastics are carbon derived from organic sources, such as microbes, lignocellulosic biomass, starch or agricultural residues, rather than fossil sources such as oil and coal. However, the term biodegradable plastics refers to the microbial conversion of polymeric materials into carbon dioxide (CO_2_) or methane (CH_4_), water, and biomass under specific environmental conditions such as soil, compost, and aqueous environments [14,15]. Importantly, these attributes are not intrinsically linked. For example, bio-derived polyethylene (Bio-PE) is biobased but not biodegradable, while certain biodegradable polymers such as polycaprolactone (PCL) and polybutylene adipate terephthalate (PBAT) are synthesised from fossil-based feedstocks. This distinction highlights that neither feedstock origin nor biodegradability alone is sufficient to define material sustainability. A holistic evaluation requires a comprehensive lifecycle perspective that accounts for broader ecological trade-offs. Upstream, biobased feedstocks can incur significant environmental costs through intensive land use, high water consumption, and fertiliser runoff. Downstream, biodegradability is highly context-dependent; most biopolymers require specialised industrial composting infrastructure to degrade properly, failing to break down efficiently in natural ecosystems.

**Figure 1:**
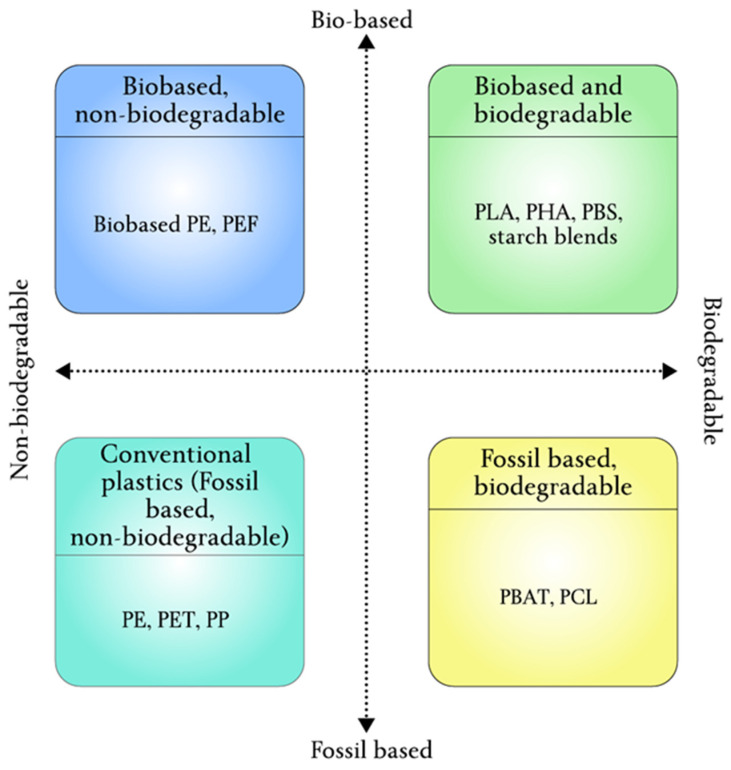
Classification of polymeric materials based on source of raw material (biobased, fossil-based) and material biodegradability (adapted from European Bioplastics [16]). Abbreviation: PE—polyethylene, PET—polyethylene terephthalate, PLA—polylactic acid, PHA—polyhydroxyalkanoate, PBS—polybutylene succinate, PP—polypropylene, PCL—polycaprolactone, PBAT—polybutylene adipate terephthalate.

Accordingly, polymeric materials can be broadly categorised into four groups, as illustrated in Figure 1 [14,16,17].

- Biobased but non-biodegradable: These include polyethylene (Bio-PE), polypropylene (Bio-PP), and polyethylene furanoate (Bio-PEF), which possess chemical structures identical to their petroleum-derived counterparts.
- Biobased and biodegradable: These include polylactic acid (PLA), polybutylene succinate (Bio-PBS), and PHB.
- Fossil-based but non-biodegradable: These include polyethylene (PE), polypropylene (PP), and polystyrene (PS).
- Fossil-based and biodegradable: These include polybutylene adipate terephthalate (PBAT) and polycaprolactone (PCL), which are conventional biodegradable polymers.

While widely adopted, this classification oversimplifies the complex relationship between material properties, processing requirements, and environmental performance. In practice, the sustainability of polymer systems depends on a combination of lifecycle impacts, functional performance, and the conditions under which degradation occurs [18,19].

Biodegradable polymers, in particular, have attracted attention due to their potential to reduce plastic waste derived from single-use and short-term disposable conventional plastics. However, the process of biodegradation depends on the environmental conditions (soil, compost and aqueous media), including temperature, inoculum and humidity, and on the material or application itself.

### 2.1. Polymer Biodegradation Process

Biodegradable polymers have attracted increasing attention due to their potential to reduce the long-term environmental persistence of plastic waste. Under suitable conditions (compost, soil and aqueous), these materials can be decomposed by microorganisms into carbon dioxide (CO_2_), water, methane (CH_4_), and biomass, making them particularly attractive for applications prone to environmental leakage, such as packaging and agricultural films.

The biodegradation process can occur through three main stages: fragmentation, hydrolysis, and microbial assimilation (mineralisation). Fragmentation involves the breakdown of high-molecular-weight polymer chains into smaller units, driven by environmental abiotic factors such as temperature, ultraviolet (UV) radiation, mechanical stress, humidity, and microbial activity as the main biotic factor [14,20]. This process increases surface area and facilitates further degradation [14,21]. Hydrolysis subsequently cleaves ester bonds within the polymer backbone, producing soluble oligomers and monomers with functional groups such as carboxyl and hydroxyl groups while also reducing molecular weight and altering material properties [14,22]. In the final stage of the mineralization process, microorganisms assimilate these low-molecular-weight compounds, converting them into CO_2_ or CH_4_, water, and biomass [14,21,23].

Despite this established pathway, biodegradation efficiency varies significantly across environments, including home composting and industrial composting systems, as well as soil, freshwater, and marine conditions [14]. Factors such as temperature, pH, moisture, oxygen availability, microbial activity, and polymer characteristics (e.g., crystallinity and molecular weight) strongly influence degradation rates [22,24]. Consequently, materials that degrade efficiently under managed industrial composting conditions may persist in natural environments indefinitely when exposed to ambient soil or aquatic environments, highlighting a key limitation in the ecological utility of biodegradable plastics. Within this context, PHAs have emerged as a distinctive class of polymers that combine biobased origin with intrinsic biodegradability, supporting their potential as sustainable material alternatives.

### 2.2. Polyhydroxyalkanoates (PHAs)

**Figure 2:**
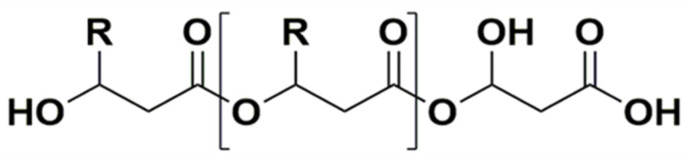
Chemical structure showing the general structure of PHA. The side-chain substituent “R” represents either a hydrogen atom or an alkyl group (e.g., methyl, ethyl, propyl, pentyl, or nonyl).

Polyhydroxyalkanoates are a class of microbially synthesised polyesters produced via metabolic pathways that convert carbon substrates into hydroxyalkanoate monomers, which are subsequently polymerised and stored as intracellular granules [2,21]. Figure 2 shows the general structure of PHAs.

A wide range of microorganisms, including species from genera such as *Cupriavidus*, *Bacillus*, and *Pseudomonas*, are capable of PHA accumulation under conditions of excess carbon and nutrient limitation (e.g., nitrogen or phosphorus) [12,20,25]. This biological production route enables the utilisation of diverse carbon sources, including sugars, fatty acids, and waste-derived substrates, highlighting the potential of PHAs as sustainable polymers.

A defining feature of PHAs is their structural diversity, which arises from variations in monomer composition and chain length. Based on monomer structure, PHAs are broadly classified as follows [2,21]:

- Short-chain-length PHAs (scl-PHAs): containing 3–5 carbon atoms per monomer unit;
- Medium-chain-length PHAs (mcl-PHAs): containing 6–14 carbon atoms;
- Long-chain-length PHAs (lcl-PHAs): containing more than 14 carbon atoms.

Short-chain-length PHAs represent the most extensively studied group and include polymers such as PHB and polyhydroxyvalerate (PHV). In contrast, medium-chain-length PHAs generally exhibit greater flexibility and elastomeric properties [20]. The variations in PHA monomer compositions are due to their carbon substrates and specificity to the producing microorganism(s). Compared to mcl-PHA monomers, scl-PHA has lower glass transition temperatures (5–9 °C) and a wider melting range (around 173–180 °C) [5,20,21]. Long-chain-length PHAs are less commonly reported but may possess unique material characteristics suitable for specialised applications.

In addition to chain length, PHAs may exist as homopolymers or copolymers. Copolymerisation is widely used to tailor thermal and mechanical properties, particularly to improve flexibility and processability [26,27]. However, such modifications often introduce trade-offs, including increased production complexity, higher costs, and, in some cases, altered biodegradation behaviour. These trade-offs highlight a central challenge in PHA development: the difficulty of simultaneously optimising material performance, processing characteristics, and economic feasibility.

The structural diversity of PHAs, largely governed by variations in side-chain substituents (Table 1), further influences crystallinity, melting temperature, glass transition temperature, and mechanical properties [28]. While this tunability is frequently cited as a key advantage, it also complicates material standardisation and large-scale processing [29]. In industrial contexts, variability in polymer composition, often linked to feedstock type and microbial strain, can result in inconsistent material properties, posing challenges for quality control and application-specific performance [17,29].

Consequently, despite their biodegradability and renewable origin, PHAs have not yet achieved widespread commercial adoption. This reflects broader limitations associated with production costs, scalability, and material performance consistency. Among the PHA family, Poly (3-hydroxybutyrate), or PHB, remains the most extensively studied homopolymer and serves as a representative system for evaluating these challenges. As such, PHB provides a critical platform for examining how structural characteristics influence production processes, material properties, and ultimately the feasibility of sustainable commercialisation, forming the primary focus of the sections that follow.

## 3. Polyhydroxybutyrate (PHB)

Polyhydroxybutyrate is the most extensively studied and commercially relevant member of the PHA family and represents one of the earliest identified microbial biopolymers. Structurally, PHB is a short-chain-length polyester composed of repeating units of (R)-3-hydroxybutyrate linked via ester bonds [21,27]. It is synthesised intracellularly by a wide range of microorganisms as a carbon and energy storage compound, typically under conditions where carbon is in excess while other essential nutrients, such as nitrogen or phosphorus, are limiting [25].

Within microbial cells, PHB accumulates as discrete intracellular granules and can constitute up to 80–90% of the cell dry weight under optimised conditions [25,30]. This exceptionally high accumulation capacity has made PHB an attractive target for industrial biopolymer production. From a materials perspective, PHB exhibits thermoplastic behaviour, is fully biodegradable, and demonstrates mechanical properties comparable to conventional polymers such as polypropylene, particularly in terms of tensile strength and stiffness [31,32].

However, despite these favourable attributes, PHB has not achieved widespread commercial penetration. Its high crystallinity results in inherent brittleness, while its narrow thermal processing window complicates conventional polymer processing [25,33]. These intrinsic material limitations, coupled with high production costs associated with microbial fermentation and downstream processing, continue to constrain its large-scale adoption.

As a result, PHB occupies a unique position within the bioplastics landscape: it is simultaneously one of the most promising and most challenged materials. Understanding this paradox requires examining not only its fundamental properties but also the historical and technological factors that have shaped its development. The following subsections therefore integrate scientific description with commercialisation context, focusing on how structural characteristics, production pathways, and historical development trajectories influence current industrial viability.

### 3.1. Historical Development of PHB: From Discovery to Commercial Constraints

Polyhydroxybutyrate was first identified in 1926 by Maurice Lemoigne, who observed intracellular polyester granules in *Bacillus megaterium* [34]. Early investigations throughout the mid-twentieth century established that PHB functions as an energy and carbon storage compound, accumulated by microorganisms under nutrient-limited conditions [25,34,35]. These foundational studies confirmed that PHB biosynthesis is a widespread metabolic capability among bacteria and can occur across a range of substrates.

From a scientific standpoint, these discoveries positioned PHB as a biologically derived analogue to synthetic thermoplastics, such as polypropylene. However, early research remained largely academic, with limited consideration of industrial scalability or economic feasibility. A significant shift occurred during the 1970s and 1980s, when the volatility of global oil markets and growing concerns over persistent plastic pollution stimulated interest in biodegradable and renewable materials [18,23,36]. This era transitioned PHB from a physiological curiosity into a target for large-scale microbial fermentation technology. However, the high cost of pure carbon substrates often accounted for up to 50% of total production costs, hindering widespread commercial adoption [37]. Significant progress was made through early industrial efforts, most notably by Imperial Chemical Industries PLC (ICI), which led to the development of the first commercialised PHB-based materials under the trade name Biopol^®^ (Imperial Chemical Industies, Millbank, London, UK). By incorporating 3-hydroxyvalerate monomers to create the copolymer PHBV, ICI addressed the inherent brittleness of pure PHB, though market penetration remained limited due to the prevailing economic competition with petroleum-based polyolefins [37,38].

Ultimately, the momentum for first-generation commercialisation efforts revealed critical limitations. Production costs were substantially higher than those of petrochemical plastics, largely due to:

- Expensive carbon substrates;
- Low volumetric productivity;
- Energy-intensive downstream recovery processes.

In parallel, material performance issues, particularly brittleness and thermal instability, limited PHB’s applicability in conventional plastic processing systems. These challenges resulted in the withdrawal or scaling down of several early commercial initiatives.

Subsequent decades have seen continued advances in metabolic engineering, fermentation optimisation, and feedstock diversification, including the use of waste-derived carbon sources. Nevertheless, the same fundamental barriers cost competitiveness and material performance have persisted, albeit in evolving forms.

This historical trajectory of PHB highlights a recurring pattern: scientific feasibility has consistently outpaced economic viability.

These economic viability challenges are closely linked to the intrinsic structure–property relationships of PHB, making it essential to examine how its molecular architecture governs performance limitations, processing behaviour, and industrial applicability.

### 3.2. Structure-Property Relationships of PHB and Implications for Processing

**Figure 3:**
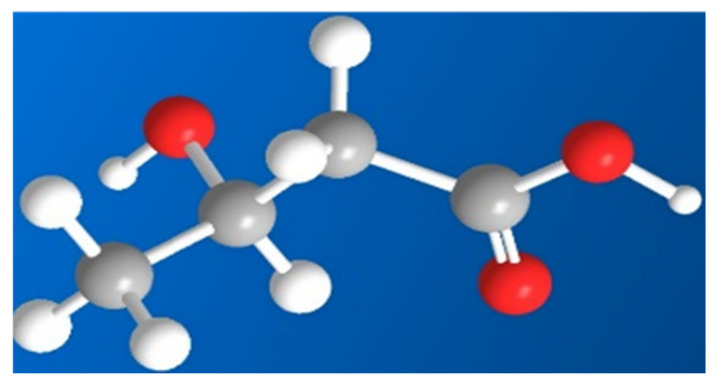
3D Structure of PHB (Chemdraw). Grey—Carbon (C), white—Hydrogen (H), red—Oxygen (O).

The performance and industrial applicability of PHB are fundamentally governed by its molecular structure and resulting physicochemical properties. PHB is a linear aliphatic polyester composed of repeating (R)-3-hydroxybutyrate units linked via ester bonds as shown in Figure 3 below [35]. The stereoregular configuration of these repeating units results in a highly ordered polymer structure, which is a defining feature underpinning both the advantages and limitations of PHB.

At the molecular level, the isotactic arrangement of PHB chains promotes strong intermolecular interactions, primarily through dipole–dipole interactions between ester functional groups [23,27,35]. This facilitates tight chain packing and leads to a high degree of crystallinity, typically exceeding 50% [33,39]. While this structural organisation contributes to relatively high stiffness and strength, comparable to conventional polymers such as polypropylene, it simultaneously restricts chain mobility, resulting in low elongation at break and inherent brittleness. A deeper understanding of the crystal structure and physical properties of PHB is therefore essential for improving its processability and expanding its range of applications.

#### 3.2.1. Crystal Structure of PHB

The crystal structure of PHB has been extensively investigated using X-ray diffraction analysis, particularly in early studies conducted on fibres produced by microbial fermentation [35,39]. These studies revealed that PHB can exist in two distinct crystalline forms: the α (alpha) form and the β (beta) form. The α form represents the thermodynamically stable crystal structure and consists of regularly packed polymer chains arranged within an orthorhombic unit cell [39]. The unit cell dimensions have been reported as a = 0.576 nm, b = 1.320 nm, and c = 0.596 nm, with a space group of P2_1_2_1_2_1_ [39]. Within this structure, two polymer chains are arranged in an antiparallel configuration, which promotes strong dipole–dipole interactions between ester groups along the polymer backbone [28,39]. These intermolecular interactions play a major role in stabilising the crystalline structure.

In contrast, the β form represents a strain-induced paracrystalline structure that forms when PHB films or fibres are subjected to mechanical stretching. This crystal form is characterised by an extended chain conformation with a twisted planar zig-zag arrangement [28,35]. Unlike the α form, the β form does not require prior alignment of the α-lamellae but instead arises from the orientation of polymer chains located within the amorphous regions between crystalline lamellae [35].

At the microscopic level, PHB crystallises in several morphological forms depending on processing conditions. Under controlled solvent crystallisation, single monolamellar crystals with well-defined structures can form in solvents such as chloroform/ethanol mixtures, propylene carbonate, and polyethylene glycol [27,39,40,41]. These crystals typically exhibit lath-shaped morphologies, with dimensions of approximately 0.3–2 μm along the short axis and 5–10 μm along the long axis, while lamellar thickness generally ranges between 4 and 10 nm, depending on molecular weight, solvent conditions, and crystallisation temperature.

In bulk materials such as films and moulded products, PHB typically forms multilamellar crystalline structures rather than isolated monolamellae [41,42]. When crystallisation occurs from the melt, spherical semicrystalline structures known as spherulites develop. These structures consist of radially growing lamellae that originate from nucleation centres within the polymer matrix [27,28]. The growth kinetics of PHB spherulites have been found to be strongly temperature-dependent, with optimal growth rates typically observed between 50 and 60 °C [27].

The formation and orientation of these crystalline structures play a critical role in determining the mechanical performance and thermal behaviour of PHB materials.

#### 3.2.2. Physical Properties of PHB

The physical properties of PHB are closely linked to its molecular weight, crystallinity, and processing conditions. In naturally occurring PHB produced by wild-type microorganisms, the molecular weight typically ranges between 1 × 10^4^ and 3 × 10^6^ g mol^−1^, with a polydispersity index of approximately two [43].

Thermally, PHB exhibits a glass transition temperature (Tg) of approximately 4 °C and a melting temperature (Tm) close to 180 °C, as determined by calorimetric analysis [28,43]. The density of PHB is approximately 1.18 g cm^−3^ in the amorphous state and increases to about 1.26 g cm^−3^ in the crystalline state [44].

Mechanically, PHB demonstrates relatively high stiffness compared with many biodegradable polymers, as shown in Table 2. Typical values reported for PHB include a Young’s modulus of approximately 3.5 GPa and a tensile strength of around 40–43 MPa. These values are comparable to those of polypropylene, indicating that PHB possesses sufficient mechanical strength for various structural applications.

However, the high crystallinity of PHB also results in poor ductility, making the material relatively brittle compared with many conventional plastics [56]. This phenomenon occurs due to the reorganisation of lamellar crystals that restrict the mobility of amorphous polymer chains [27]. To mitigate this issue, various strategies have been explored, including annealing treatments like reinforcement of organic and inorganic fillers that promote controlled crystallisation and reduce internal stresses within the polymer matrix [55,71].

Advances in microbial biotechnology have also enabled the production of ultra-high molecular weight PHB through genetic engineering approaches. For example, the cloning of PHB biosynthesis genes from *Ralstonia eutropha* into *Escherichia coli* has enabled the production of PHB with molecular weights ranging from 3 × 10^6^ to 1.1 × 10^7^ g mol^−1^ under optimised fermentation conditions [72,73]. Materials produced from such ultra-high molecular weight PHB exhibit improved mechanical properties and can be processed into stretched films with enhanced strength and stiffness.

Numerous studies [5,33,74,75] have shown that stretched PHB films can achieve Young’s modulus values of approximately 1.1 GPa and tensile strengths of up to 62 MPa, significantly improving performance compared with unstretched materials. Furthermore, additional annealing treatments applied to stretched films can further enhance mechanical performance by promoting improved crystalline organisation [76].

These developments demonstrate that biotechnological approaches and controlled processing techniques can significantly improve the material properties of PHB, thereby enhancing its potential for large-scale commercial applications.

Although manipulation of these crystalline structures through processing (e.g., stretching or annealing) can improve mechanical performance, such approaches introduce additional processing steps and costs, which may not be economically viable at large scale.

The structure–property relationships of PHB therefore present a series of inherent trade-offs. High crystallinity enhances strength and barrier properties but compromises flexibility and toughness. Similarly, while stereoregularity supports desirable mechanical characteristics, it contributes to thermal instability during processing. These competing effects highlight a fundamental limitation: the intrinsic molecular architecture of PHB is not optimally balanced for conventional plastic processing and application requirements.

While the intrinsic structure–property relationships of PHB impose significant limitations on processing and performance, these challenges are further compounded by constraints in its production systems. A comprehensive evaluation of PHB’s commercial viability therefore requires examination of the bioprocessing pathways, feedstock requirements, and cost structures that govern its large-scale manufacture.

### 3.3. Production of PHB: Bioprocesses, Feedstocks, and Cost Constraints

Despite the favourable biodegradability and mechanical properties of PHB, its large-scale adoption remains fundamentally constrained by production-related challenges. Unlike conventional plastics derived from well-established petrochemical processes, PHB is synthesised through microbial fermentation, introducing additional layers of biological, engineering, and economic complexity. While significant advances have been made in improving yields and process efficiency, the interdependence between microbial metabolism, feedstock selection, downstream recovery, and overall production cost continues to limit commercial competitiveness. This section critically examines the key stages involved in PHB production, highlighting the trade-offs between process efficiency, material quality, and economic feasibility.

#### 3.3.1. Microbial Biosynthesis of PHB

Polyhydroxybutyrate is synthesised intracellularly by numerous microorganisms as a carbon and energy storage polymer, typically under conditions of excess carbon and limitation of essential nutrients such as nitrogen or phosphorus. Under optimised conditions, specific microorganisms, both native and genetically engineered, can accumulate PHB up to 80–90% of their cell dry weight (CDW), highlighting the intrinsic efficiency of the biological system [77,78]. Among these, *Cupriavidus necator* remains the most widely studied and industrially relevant organism, capable of achieving PHB contents approaching 93% (*w*/*w*) when cultivated on substrates such as vegetable oils [79]. Other organisms, including *Bacillus thuringiensis* [80], *Halomonas boliviensis* [81,82], and *Paraburkholderia sacchari* [83,84], have also demonstrated relatively high PHB yields (typically 40–70%) across a range of carbon sources, including sugarcane-derived substrates, starch hydrolysates, and simple sugars.

The diversity of PHB-producing microorganisms is reflected in their ability to utilise a wide spectrum of carbon feedstocks as highlighted above. This ranges from refined substrates such as glucose and sucrose to more complex and low-cost alternatives including methanol, xylose, plant oils, and agro-industrial residues. For example, methylotrophic bacteria such as *Methylobacterium* spp. can synthesise PHB using methanol [85], while organisms such as *Azotobacter vinelandii* [86] and *Serratia* spp. [87] have demonstrated PHB production from lignocellulosic derivatives such as wheat bran and xylose, respectively. This metabolic versatility presents a significant opportunity for integrating waste-derived or regionally abundant feedstocks into PHB production systems, particularly in resource-constrained contexts.

However, this apparent flexibility also introduces variability in both process performance and polymer characteristics. Differences in microbial metabolism and substrate composition can influence not only PHB yield but also molecular weight distribution, crystallinity, and thermal behaviour of the resulting polymer [88]. As a result, achieving consistent material properties suitable for industrial processing remains a challenge when transitioning between feedstocks or microbial systems.

A further limitation arises from the metabolic conditions required to induce PHB accumulation. While nutrient limitation promotes polymer synthesis, it simultaneously suppresses cell growth, leading to reduced volumetric productivity [89,90]. This creates a fundamental trade-off between biomass generation and polymer accumulation, complicating fermentation strategies and scale-up. In practice, multi-stage or fed-batch processes are often required to balance these competing demands, increasing operational complexity.

In addition to upstream variability, the intracellular nature of PHB introduces inherent challenges in downstream recovery. The polymer must be extracted from within the microbial cell, and the choice of extraction method significantly affects both polymer purity and molecular integrity. These methods range from solvent-based techniques using chloroform or cyclohexanone to chemical digestion (e.g., sodium hypochlorite) or enzymatic disruption [91]. While solvent extraction methods typically yield high-purity PHB, they are associated with high costs, environmental concerns, and solvent recovery requirements. Conversely, non-solvent or biological extraction approaches may reduce environmental impact but often result in lower purity or polymer degradation.

From a commercialisation perspective, the breadth of microbial systems, substrates, and recovery methods reflects both the potential and the complexity of PHB production. While high yields can be achieved under controlled laboratory conditions, the combined effects of metabolic trade-offs, feedstock variability, and extraction challenges limit the translation of these systems into robust, large-scale processes. Consequently, microbial biosynthesis, although fundamentally viable, remains tightly coupled to downstream processing constraints and overall production economics, necessitating integrated optimisation across the entire value chain. This diversity in microbial systems, substrates, and recovery methods is summarised in Table 3, which highlights the inherent trade-offs between yield, feedstock flexibility, and downstream processing requirements.

While high-yield strains and diverse feedstocks demonstrate the technical feasibility of PHB production, the interplay between substrate choice, recovery method, and material purity introduces trade-offs that directly influence process economics and scalability. These constraints highlight that maximising yield alone is insufficient; rather, an integrated optimisation of upstream and downstream parameters is required.

**Figure 4:**
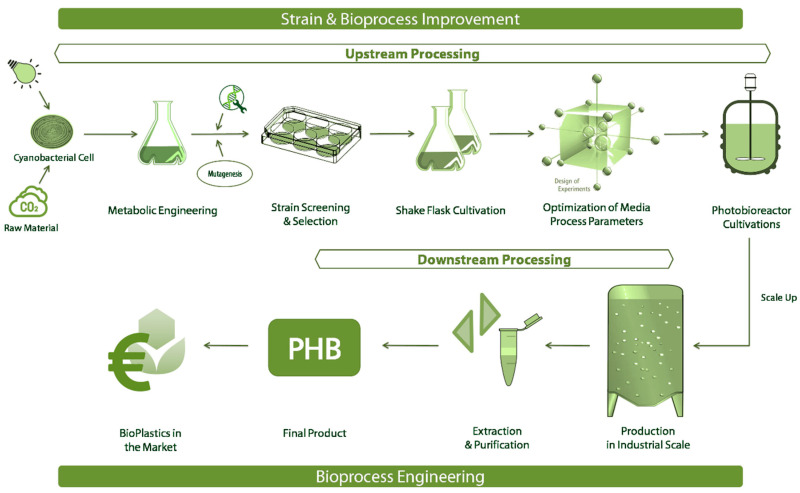
Integrated bioprocess workflow for PHB production. Illustrating upstream strain and fermentation optimisation together with downstream extraction, purification, scale-up, and product development pathways. (Reproduced from Kamravamanesh et al. [94] under the CC BY 4.0 licence).

The transition from microbial PHB biosynthesis to commercially relevant production requires integration of upstream cultivation, downstream recovery, and process engineering strategies. Figure 4 illustrates the general bioprocess workflow involved in PHB production, from strain improvement and cultivation to polymer extraction, purification, and product development.

This necessitates a closer examination of these components including fermentation conditions and bioprocess design, where upstream processing constraints play a decisive role in determining productivity, consistency, and ultimately, the commercial viability of PHB systems.

#### 3.3.2. Upstream Processing Constraints: Feedstocks, Fermentation Strategies and Cost Drivers

Upstream processing remains one of the most significant barriers to the industrial production and commercial competitiveness of PHB. Although laboratory-scale systems frequently demonstrate high polymer yields and efficient microbial accumulation, these results are often difficult to reproduce under industrial conditions due to the interdependence of biological performance, reactor engineering, and process economics. The integrated nature of PHB production is illustrated in Figure 4, where upstream processing encompasses optimal nutrient conditions, strain improvement, microbial screening, media optimisation, and bioreactor cultivation. These stages collectively determine cell density, polymer accumulation, substrate utilisation efficiency, and overall production productivity.

A central challenge in PHB biosynthesis is the requirement for nutrient-limited conditions, typically nitrogen or phosphorus limitation, in the presence of excess carbon to induce intracellular polymer accumulation [89,95]. While these conditions stimulate PHB synthesis, they are inherently suboptimal for microbial growth, creating a critical trade-off between maximising cell biomass and maximising intracellular polymer accumulation. High cell density is required to improve volumetric productivity and overall process economics; however, excessive nutrient limitation can suppress biomass formation, whereas nutrient excess promotes growth at the expense of polymer accumulation [3,31]. Industrial fermentation therefore requires careful balancing of these competing metabolic objectives, often through fed-batch or multi-stage cultivation strategies, which increase process complexity and operational costs.

Substrate selection is another major determinant of upstream economics, with carbon feedstocks accounting for approximately 40–60% of total PHB production costs [96,97]. Refined substrates such as glucose and sucrose provide high fermentation consistency and predictable polymer quality but are economically unfavourable at scale [21]. In contrast, lower-cost alternatives including lignocellulosic hydrolysates, crude glycerol [12], waste oils [79], and agro-industrial residues [80] offer improved economic potential but introduce substrate heterogeneity, inhibitory compounds, and pretreatment requirements, respectively. As shown in Table 3, microorganisms such as *Cupriavidus necator* can achieve PHB yields as high as 93% under optimised conditions using refined lipid substrates, whereas systems based on alternative or waste-derived substrates often exhibit lower or more variable yields [12,58,79]. This reflects a recurring commercial trade-off in PHB production: substrate cost reduction is frequently accompanied by reduced process stability or productivity.

Microbial strain selection further influences both technical performance and process economics. *Cupriavidus necator* remains the benchmark strain for high-yield PHB production, but its cultivation generally requires sterile conditions, controlled aeration, and tightly regulated environmental parameters [79,98]. These requirements increase capital and operational expenditure through sterilisation, monitoring, and contamination control. Alternative strains such as halophilic *Halomonas* species have attracted interest due to their ability to grow under hypersaline and potentially non-sterile conditions, thereby reducing contamination risk and sterilisation costs [96]. Similarly, recombinant systems such as engineered *Escherichia coli* offer metabolic flexibility but may suffer from reduced polymer yields or require expensive induction and downstream purification steps [97]. Collectively, these examples demonstrate that no single microbial platform currently satisfies the combined requirements of high productivity, low contamination risk, operational simplicity, and cost efficiency.

At industrial scale, reactor engineering introduces further constraints. PHB-producing microorganisms are predominantly aerobic, making oxygen availability a major limiting factor in high-density fermentations. As reactor volume increases, oxygen transfer becomes increasingly difficult due to reduced gas–liquid mass transfer efficiency, higher broth viscosity, and insufficient mixing [99]. Oxygen limitation can suppress microbial metabolism and reduce PHB productivity, necessitating increased aeration and agitation rates, which substantially elevate energy consumption. In parallel, large-scale systems are highly sensitive to environmental fluctuations including pH, temperature, dissolved oxygen, and nutrient feed rates, all of which require precise control to maintain consistent polymer quality and yield.

Contamination risk remains an additional operational challenge, particularly in conventional fermentation systems using nutrient-rich media [100]. The need to maintain aseptic conditions increases sterilisation demands and limits the economic attractiveness of certain microbial platforms. Although extremophilic organisms partially address this limitation, their industrial implementation remains comparatively underdeveloped [101].

These technical challenges translate directly into unfavourable production economics. Current PHB production costs are estimated at approximately USD 6–8 kg^−1^ for conventional fermentation systems, with optimised processes using low-cost or waste-derived substrates reducing costs to approximately USD 3–5 kg^−1^ under favourable conditions [7,102]. By comparison, conventional fossil-derived plastics such as polypropylene and polyethylene are typically produced at approximately USD 0.80–1.60 kg^−1^, making them more than 2–8 times cheaper than PHB, depending on process configuration and market conditions [7,103]. This substantial cost disparity remains one of the most persistent barriers to PHB market penetration despite its favourable biodegradability profile.

Taken together, these findings indicate that upstream optimisation alone cannot resolve the commercial limitations of PHB. Improvements in microbial strain engineering, substrate diversification, or fermentation design may reduce production costs incrementally, but the cumulative burden of feedstock costs, process sensitivity, oxygen transfer limitations, contamination management, and environmental control requirements continues to constrain scalability and cost competitiveness.

However, beyond microbial physiology and reactor optimisation, the economic feasibility of PHB production is also heavily influenced by the selection of carbon substrates used during fermentation. Feedstocks not only determine microbial growth and polymer accumulation efficiencies but also contribute substantially to overall production costs, downstream processing complexity, and the environmental sustainability of the process. As a result, substrate selection has become a central consideration in efforts to improve the commercial competitiveness of PHB production systems.

#### 3.3.3. Feedstock and Substrate Selection

While optimisation of microbial strains and fermentation conditions remains important, the economics of PHB production are also strongly influenced by the choice of carbon substrate. Feedstocks typically account for a substantial proportion of total production costs, with estimates suggesting that carbon sources alone may contribute between 30 and 50% of overall operating expenses in large-scale PHB production systems [45,104]. Consequently, substrate selection has emerged as a critical factor influencing not only polymer yield and productivity but also process sustainability and commercial feasibility. As shown in Figure 4, feedstock selection forms a central component of upstream bioprocess engineering because the choice of carbon substrate directly influences microbial growth kinetics, PHB yield, downstream purification requirements, and overall process economics.

A wide range of carbon substrates has been investigated for PHB synthesis as shown in Table 4, including refined sugars, plant oils, organic acids, lignocellulosic hydrolysates, industrial by-products, wastewater-derived carbon streams and C_1_ substrate sources.

Conventional substrates, such as glucose and sucrose, support high microbial growth rates and efficient PHB accumulation; however, their high cost limits economic competitiveness against petroleum-derived plastics. This economic barrier has driven interest in lower-cost, waste-derived feedstocks that reduce substrate expenditure while improving environmental sustainability.

Among the most promising alternatives are agricultural and agro-industrial residues:

- Agro-industrial By-products: Molasses, cheese whey, and starch hydrolysates offer excellent opportunities for biorefinery integration [11].
- Crude Glycerol: A major byproduct of biodiesel production [12].
- Lignocellulosic Biomass: Highly abundant and renewable, making it a viable long-term option for agricultural economies [10].
- Lipid-Rich Waste Streams: Waste cooking oils and food-processing wastes are highly attractive due to their high carbon density and non-competition with food resources [80,106].

Several microorganisms, including *Cupriavidus necator* [12,79], *Halomonas boliviensis* [81,82], and *Burkholderia sacchari* [95], have demonstrated robust capabilities to accumulate substantial PHB yields using these complex substrates.

While economically attractive, utilising waste-derived feedstocks introduces significant operational and metabolic complexities:

- Pretreatment Inhibitors: Lignocellulosic biomass requires energy-intensive pretreatment and hydrolysis to release fermentable sugars. These processes frequently generate microbial inhibitors, such as furfurals, weak organic acids and phenolics, which impair cell growth and PHB polymer synthesis [3,111]. Consequently, detoxification strategies such as overliming, activated carbon adsorption, ion-exchange resins, membrane separation, and biological detoxification are frequently employed to improve hydrolysate fermentability [112]. However, these additional processing steps increase both energy demand and operating costs, reinforcing pretreatment as one of the principal technoeconomic bottlenecks in lignocellulosic PHB production. Improving pretreatment efficiency, reducing chemical consumption, and integrating heat recovery therefore remain key priorities for enhancing the overall technoeconomic feasibility of lignocellulosic PHB production. Despite these challenges, continued advances in enzymatic hydrolysis, consolidated bioprocessing, and robust microbial strains capable of co-fermenting mixed sugars are expected to improve the commercial viability of second-generation feedstocks.
- Mass Transfer Limitations: Lipid-based substrates exhibit high hydrophobicity, causing severe mass transfer limitations that reduce bioavailability unless specialised mixing or emulsification strategies are applied [31,113].
- Impurity and Compositional Volatility: Industrial by-products like crude glycerol and molasses suffer from variable composition and impurity profiles. For instance, residual methanol and salts in crude biodiesel glycerol inhibit microbial metabolism unless mitigated by upstream purification or strain adaptation [31].
- Process Reproducibility: Wastewater-derived carbon streams present extreme composition fluctuations and often necessitate mixed microbial cultures (MMCs), which can compromise process reproducibility and final polymer uniformity [109].

Beyond terrestrial wastes, emerging third- and fourth-generation feedstocks (C1 gases and algae), including methanol, methane, syngas, and carbon dioxide, decouple PHB production from agricultural land use while enabling greenhouse gas utilisation [110]. Nevertheless, these gas fermentation systems face distinct engineering challenges, including poor gas–liquid mass transfer, lower volumetric productivity, and stringent operational safety requirements regarding flammable or toxic gases [110,114].

Whilst upstream feedstock selection can be narrowed down to meet specific requirements, this is not the case for downstream processing, as impurities originating from complex waste streams directly impact polymer recovery and quality. Inconsistent nutrient profiles and crude contaminants often necessitate additional sterilisation, detoxification, or supplementation steps, partially offsetting the initial economic advantages of the substrate [103,104]. Furthermore, these impurities can complicate extraction, lower final purity, and alter the molecular weight distribution and thermal stability of the polymer [115].

Ultimately, feedstock selection represents a critical balancing exercise between cost reduction, process stability, polymer quality, and sustainability. Although waste-derived substrates offer considerable promise for reducing production costs and improving the environmental profile of PHB, their integration into industrial-scale bioprocesses remains technically challenging. This highlights the broader reality that low-cost substrates alone are insufficient to ensure economically viable PHB production unless accompanied by robust and scalable downstream recovery systems.

Accordingly, downstream processing has emerged as another major bottleneck in the PHB value chain, with polymer extraction and purification frequently contributing substantially to overall production costs and environmental burdens.

#### 3.3.4. Downstream Processing Constraints and Commercialisation Implications

While significant advances have been achieved in microbial strain development and upstream fermentation optimisation, downstream processing remains one of the largest economic and technical bottlenecks limiting large-scale PHB commercialisation. Following intracellular accumulation, PHB must be recovered, purified, compounded, and converted into usable products while maintaining polymer quality and minimising degradation. As shown in Figure 4, these stages contribute substantially to the overall production cost of PHB and strongly influence its final material performance. In many production systems, downstream processing alone may account for a considerable proportion of total manufacturing costs due to solvent consumption, energy requirements, purification complexity, and product losses during recovery [103,116].

**Figure 5:**
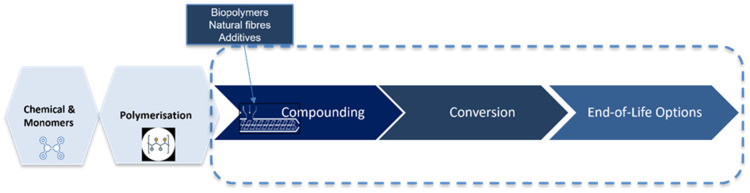
PHB downstream processing and conversion pathway showing compounding, conversion, and end-of-life stages.

The downstream value chain of PHB extends beyond simple polymer extraction and includes multiple interconnected stages, including purification, compounding, conversion into finished products, and end-of-life management, as illustrated in Figure 5. The figure highlights how PHB commercialisation depends not only on successful biosynthesis but also on the integration of material formulation and manufacturing processes capable of producing application-specific products at industrial scale.

A major challenge during downstream processing arises from the intracellular nature of PHB accumulation. Since PHB granules are stored within microbial cells, efficient cell disruption is required prior to polymer recovery. Conventional extraction methods frequently rely on chlorinated solvents such as chloroform due to their high extraction efficiency and ability to produce relatively pure polymer fractions [117]. However, these solvents are expensive, environmentally hazardous, difficult to recycle, and unsuitable for sustainable large-scale manufacturing. Residual solvent contamination also presents concerns for food-contact and biomedical applications. Consequently, solvent extraction processes often conflict with the environmental sustainability objectives that motivate PHB development in the first place.

Alternative extraction approaches, including enzymatic digestion, surfactant-assisted recovery, chemical digestion, and non-solvent precipitation methods, have therefore attracted increasing interest. Enzymatic extraction methods can preserve molecular weight and minimise polymer degradation, but their high reagent costs and relatively slow processing times limit industrial applicability [73,117]. Chemical digestion methods using sodium hypochlorite along with surfactants can simplify biomass removal, although excessive oxidation may reduce polymer molecular weight and negatively affect mechanical performance [117,118]. Similarly, non-solvent precipitation techniques may reduce solvent usage but often compromise polymer purity or recovery efficiency [119]. These trade-offs demonstrate that downstream optimisation is not solely a purification problem, but rather a balance between recovery yield, polymer quality, environmental sustainability, and economic feasibility.

Beyond extraction, PHB also presents challenges during melt processing and product conversion. Due to its narrow thermal processing window, PHB can undergo thermal degradation at temperatures near its melting point, resulting in chain scission, reduced molecular weight, and deterioration of mechanical properties during extrusion or injection moulding [115,120]. This processing instability complicates industrial manufacturing and limits compatibility with conventional plastic processing infrastructure. As a result, additional stabilisation strategies, plasticisers, blending agents, or compounding additives are often required prior to conversion into commercial products [115].

The compounding and conversion stages therefore play a critical role in determining the ultimate functionality and market applicability of PHB materials. As illustrated in Figure 4, PHB is frequently combined with other biopolymers, natural fibres, or additives to improve flexibility, toughness, thermal stability, and processing behaviour before being converted into films, packaging materials, or moulded products. However, each additional modification step increases process complexity and manufacturing costs, while potentially affecting biodegradability and recyclability. Consequently, improvements in material performance are often accompanied by new economic and sustainability trade-offs.

From a commercialisation perspective, downstream processing represents a critical intersection between biotechnology and polymer engineering. Even when high PHB yields are achieved during fermentation, inefficient recovery processes and poor melt-processability can significantly reduce industrial competitiveness. These constraints highlight an important reality within the PHB value chain: successful commercialisation depends not only on biological production efficiency but also on the development of scalable, cost-effective, and environmentally sustainable downstream processing technologies capable of producing high-performance materials compatible with existing manufacturing systems.

This growing recognition has shifted recent research efforts toward integrated bioprocess engineering approaches that simultaneously optimise upstream production, downstream recovery, material formulation, and product conversion. Such strategies are increasingly viewed as essential for improving the overall economic viability and industrial scalability of PHB-based materials.

#### 3.3.5. Technoeconomic Feasibility of PHB Production

Despite significant advances in microbial strain development, feedstock utilisation, and polymer recovery technologies, the commercial adoption of PHB remains constrained by economic considerations. While PHB offers compelling environmental advantages, including complete biodegradability and renewable production pathways, its market penetration remains limited compared with both conventional petrochemical plastics and competing bioplastics such as PLA. Consequently, technoeconomic feasibility has emerged as a critical determinant of whether PHB can transition from a niche specialty material to a large-scale commodity polymer.

The most significant commercial challenge facing PHB is its inability to consistently achieve cost parity with conventional plastics. The market price of commodity petrochemical polymers such as polypropylene (PP) and polyethylene (PE) generally ranges between USD 1.0 and 2.0 kg^−1^, whereas the reported market price of PHB generally ranges between USD 3.5 and 11.0 kg^−1^ under current industrial conditions [121,122]. In Table 5, the cost ranges of conventional plastics are compared to the current market price range of PHB.

In some cases, costs may be reduced through waste-derived feedstocks and process integration strategies, but large-scale economic competitiveness remains difficult to achieve. Several technoeconomic studies have demonstrated the potential for cost reductions through process optimisation [115,121,122,123,132,133]. Increasing intracellular PHB accumulation significantly lowers downstream processing costs because a greater proportion of harvested biomass consists of the target polymer. For example, Pavan et al. [133] showed that increasing PHB content within microbial cells reduced estimated production costs from approximately USD 4.28 kg^−1^ to USD 3.50 kg^−1^, while improvements in biomass concentration further reduced overall costs by nearly 18%.

Studies evaluating waste-derived substrates have reported even lower upstream production costs. Using *Cupriavidus necator*, estimated gate-to-gate production costs were reported at approximately USD 1.18 kg^−1^ when waste cooking oil was used and as low as USD 0.36 kg^−1^ when crude glycerol served as the primary carbon source [12,79]. However, these values exclude downstream recovery, purification, and capital costs, which remain significant contributors to the final product price.

The collective evidence suggests that PHB commercialisation is technically feasible but remains economically sensitive to feedstock selection, fermentation performance, downstream recovery efficiency, and production scale. No single technological breakthrough is likely to eliminate the cost gap between PHB and petrochemical plastics. Instead, meaningful cost reductions will require integrated improvements across the entire value chain.

This, along with preceding sections, demonstrates that the challenges facing PHB commercialisation are not solely related to production economics. Even if manufacturing costs are reduced through improved fermentation processes and low-cost feedstocks, the inherent structure–property relationships of PHB continue to limit its performance in many conventional plastic applications. As a result, considerable research has shifted towards modifying PHB at the molecular and material levels to address these performance constraints while retaining its biodegradability and biobased origin.

### 3.4. Modification Strategies for Overcoming PHB Commercialisation Constraints

The preceding sections demonstrated that the commercial limitations of PHB arise from both production economics and inherent material properties. Even when produced efficiently, PHB remains constrained by high crystallinity, brittleness, low impact resistance, and thermal instability during melt processing. These characteristics restrict its applicability in many packaging, consumer goods, and engineering applications where flexibility, toughness, and processing robustness are required. Consequently, extensive research has focused on modifying PHB through copolymerisation, polymer blending, plasticisation, and composite formation. The primary objective of these approaches is not only to improve material performance but also to expand the range of commercially viable applications and enhance the competitiveness of PHB relative to established petroleum-derived plastics.

#### 3.4.1. Copolymerisation Strategies: PHBV as the Benchmark PHB Copolymer

The need for copolymerization originates from the intrinsic molecular architecture of PHB. As discussed in Section 3.2, PHB is a highly stereoregular and semicrystalline polymer, typically exhibiting crystallinity values exceeding 50%. While this molecular organisation contributes to relatively high stiffness and tensile strength comparable to polypropylene, it also results in pronounced brittleness, low elongation at break, and limited impact resistance. Furthermore, PHB possesses a narrow processing window because thermal degradation begins at temperatures close to its melting temperature (approximately 175–180 °C) [134]. Secondary crystallisation during storage can further increase brittleness over time, creating challenges for both processing and end-use performance [27]. These limitations have motivated the development of copolymers designed to disrupt crystal packing and improve the balance between strength, flexibility, and processability.

These copolymers include poly (3-hydroxybutyrate-co-3-hydroxyvalerate) (PHBV), with randomly arranged 3-hydroxybutyrate (HB) and 3-hydroxyvalarate (HV) groups. PHBV is a biopolymer with the following properties, (1) high crystallinity with a melting point of 180 °C, (2) glass transition temperature (Tg) of the polymer is in the range −5 °C to 20 °C (3) the flexibility and processability of PHBV is higher than PHB, and (4) Increasing HV content significantly modifies the properties of PHBV by reducing crystallinity and transforming the rigid and brittle PHB homopolymer into a tougher, more flexible, and easier-to-process material. [8,28]. Modification of PHBV can be influenced by valerate content, which affects the crystallinity, melting point and rate of crystallisation of the copolymer [28,56], whereas the copolymer melting point, glass transition temperature and crystallinity decrease as the hydroxyvalerate unit content increases. With an increase of the HV units in the copolymer, the impact strength increases and the tensile strength decreases [135].

It was shown that the rate of degradation of PHBV is faster than that of PHB. The degradation kinetics were found to depend on the structure (copolymer or homopolymer), crystallinity, and processing conditions [28]. Similarly, PHBV has a heat deflection temperature like that of PP and an acceptable impact strength [56,62].

The commercial significance of PHBV lies in its ability to address several of the major performance limitations associated with PHB. Increasing hydroxyvalerate content generally reduces crystallinity and melting temperature while improving ductility and impact resistance [50]. These improvements broaden the processing window and facilitate manufacturing using conventional polymer processing technologies. However, these benefits are accompanied by trade-offs. Higher hydroxyvalerate incorporation often reduces tensile strength and may increase production costs because precursor substrates required for hydroxyvalerate synthesis are typically more expensive than those used for PHB production [28,50]. Consequently, PHBV represents a compromise between performance enhancement and economic viability rather than a complete solution to the limitations of PHB.

#### 3.4.2. Polymer Blending Approaches

Unlike copolymerisation, which modifies polymer structure during biosynthesis, polymer blending represents a downstream strategy in which PHBV is physically combined with other polymers to achieve targeted material properties. Blending offers several practical advantages, including compatibility with existing compounding infrastructure, flexibility in formulation design, and the potential to reduce overall material costs [3,55]. As a result, blending has become one of the most widely adopted approaches for addressing the remaining performance and economic limitations of PHBV.

A major objective of blending is to balance the competing requirements of stiffness, toughness, processability, biodegradability, and cost. Different blending components are therefore selected to address specific limitations of PHBV as shown in Table 6. For example, poly(lactic acid) (PLA) is frequently incorporated to improve rigidity, tensile strength, and dimensional stability [134]. However, because both PHBV and PLA are relatively brittle polymers, improvements in stiffness are often accompanied by limited gains in toughness [13,134].

To overcome this limitation, flexible biodegradable polymers such as poly(butylene adipate-co-terephthalate) (PBAT) are commonly incorporated as toughening agents [3,13]. Numerous studies have demonstrated that PHBV/PBAT blends exhibit substantially improved elongation at break, impact resistance, and toughness compared with neat PHBV [136,137,139,140]. These improvements are generally attributed to reduced crystallinity and enhanced energy dissipation during deformation. However, they also show that increased flexibility is often accompanied by reductions in tensile strength and modulus, highlighting the trade-off between toughness and structural rigidity.

Economic considerations have also driven interest in blending PHBV with lower-cost materials such as thermoplastic starch (TPS). Starch-based components can significantly reduce formulation costs while maintaining biodegradability, making such blends attractive for disposable packaging and short-life applications. Nevertheless, TPS-containing blends may exhibit increased moisture sensitivity and reduced mechanical stability, which can limit their suitability for more demanding applications [138].

Beyond binary polymer blends, researchers have explored the incorporation of natural fibres, recycled wood fibres, nanoclays, and other reinforcing fillers to compensate for losses in stiffness and strength [13,141]. These composite systems aim to create materials that combine the toughness benefits of blending with the structural performance required for practical applications.

From a commercialisation perspective, polymer blending represents a more flexible and economically attractive strategy than copolymerisation alone. However, the fundamental challenge remains unchanged: improvements in one property are frequently accompanied by compromises in another. Consequently, the development of commercially viable PHBV-based materials increasingly relies on the careful optimisation of blend composition and composite design to achieve an acceptable balance between performance, sustainability, processability, and cost.

#### 3.4.3. Plasticisers and Additives

While copolymerisation and polymer blending can substantially improve the toughness and processability of PHB, these approaches often involve additional synthesis steps, compatibility challenges, or increased material costs. Consequently, considerable attention has been directed towards the use of plasticisers and functional additives as a simpler and more cost-effective strategy for modifying PHB properties. Unlike copolymerisation, which alters the polymer structure, or blending, which introduces a secondary polymer phase, plasticisers act by increasing chain mobility within the PHB matrix, thereby reducing intermolecular interactions and enhancing flexibility [3].

The need for plasticisation arises from the inherently high crystallinity and strong intermolecular interactions of PHB, which contribute to its brittleness and low elongation at break. The incorporation of suitable plasticisers reduces the glass transition temperature (T_g_) and improves chain mobility within the amorphous regions of the polymer [3,142]. As a result, PHB materials generally exhibit improved ductility, impact resistance, and processability during extrusion and injection moulding [142].

A wide range of plasticisers have been investigated for PHB, including citrate esters, polyethylene glycol (PEG), glycerol derivatives, epoxidised vegetable oils, oligomeric lactic acid, and other biobased additives. Among these, PEG and citrate-based plasticisers have received particular attention due to their compatibility with biodegradable polymer systems and their ability to significantly improve elongation at break while reducing brittleness [143]. Similarly, epoxidised soybean oil and other renewable plasticisers have emerged as attractive alternatives because they align with the sustainability objectives associated with PHB-based materials [144].

Despite these advantages, plasticisation introduces important trade-offs. Excessive plasticiser loading can reduce tensile strength, modulus, thermal stability, and barrier performance [3,143]. Plasticiser migration during storage may also occur, resulting in progressive deterioration of material properties over time [145]. In food packaging and biomedical applications, additive migration may further raise regulatory and safety concerns. Consequently, optimisation of plasticiser type and concentration remains essential to achieve a balance between flexibility and long-term performance.

Beyond conventional plasticisers, numerous additives have been explored to enhance specific functionalities of PHB. These include nucleating agents to control crystallisation behaviour [146], thermal stabilisers to reduce degradation during processing [147], chain extenders to improve molecular weight retention [148], antioxidants to enhance storage stability [149], and compatibilisers for multiphase blend systems [150]. Such additives can improve processing performance and material consistency without fundamentally altering the polymer chemistry.

From a commercialisation perspective, plasticisation and additive incorporation represent some of the most economically accessible modification strategies available for PHB. They can often be implemented using existing polymer processing infrastructure and require relatively low capital investment compared with microbial strain engineering or copolymer synthesis [125,130]. However, these approaches do not address the underlying economic challenge associated with PHB production costs and therefore serve primarily as performance-enhancement tools rather than complete solutions to commercial competitiveness. Consequently, while plasticisers and additives can expand the application range of PHB, they are most effective when integrated with broader strategies involving copolymerisation, blending, and process optimisation. Research has increasingly focused on the incorporation of reinforcing fillers and nanomaterials capable of simultaneously enhancing stiffness, strength, thermal stability, and barrier properties. These reinforcement strategies are discussed in the following section.

#### 3.4.4. Reinforcement Fillers and Nanocomposite Approach

While plasticisers improve flexibility and processing behaviour, they often reduce stiffness and strength as discussed earlier. Consequently, considerable research has focused on the incorporation of reinforcing fillers and nanomaterials to enhance the mechanical, thermal, and barrier properties of PHB without sacrificing structural performance. These reinforcement strategies represent another important approach for overcoming the inherent limitations associated with the high crystallinity and brittleness of PHB.

Conventional fillers such as cellulose fibres, wood flour, lignin, talc, calcium carbonate, and natural fibres have been incorporated into PHB matrices to improve stiffness, dimensional stability, and cost competitiveness [151]. Among these, lignocellulosic fillers have attracted particular interest because they are renewable, inexpensive, and compatible with the sustainability objectives of bioplastic production [137,152]. The incorporation of agricultural residues and natural fibres can simultaneously reduce polymer consumption and improve the biobased content of the final material.

More recently, nanoscale reinforcements have been investigated to achieve greater property enhancements at relatively low filler loadings. Common nanomaterials explored in PHB systems include nanoclays, cellulose nanocrystals (CNCs), cellulose nanofibres (CNFs), graphene derivatives, carbon nanotubes, and silica nanoparticles [153,154]. Due to their high surface area and strong interfacial interactions with the polymer matrix, these materials have been reported to significantly improve tensile strength, stiffness, thermal stability, and gas barrier performance [3,154].

Numerous studies have demonstrated that nanoclay incorporation can improve PHB stiffness and reduce gas permeability, making such materials attractive for food packaging applications [141,155,156]. Similarly, cellulose nanocrystals and nanofibres have shown promise as sustainable reinforcement agents capable of improving mechanical performance while maintaining biodegradability [113,157]. Carbon-based nanomaterials can provide even greater improvements in mechanical and electrical properties, although their relatively high cost and sustainability concerns currently limit widespread commercial implementation [3,158].

Despite these promising results, reinforcement strategies introduce their own technical challenges. Achieving uniform dispersion of fillers within the PHB matrix remains difficult due to particle agglomeration and incompatibility between hydrophilic fillers and the relatively hydrophobic polymer phase [137,156]. Poor dispersion can create stress concentration points that reduce rather than improve mechanical performance. As a result, additional processing steps, surface treatments, or compatibilisers are frequently required to achieve effective reinforcement [71].

Commercially, reinforcing fillers present a mixed opportunity. Low-cost natural fillers may reduce overall material costs while improving selected properties, particularly in packaging and disposable product applications. In contrast, high-performance nanomaterials often increase formulation complexity and manufacturing costs. Furthermore, the economic benefits gained through improved mechanical performance may not fully offset the additional costs associated with filler processing, dispersion technologies, and quality control.

Consequently, reinforcement strategies should be viewed as performance-enhancement tools rather than standalone solutions to PHB commercialisation challenges. While fillers and nanocomposites can address several material limitations, they do not fundamentally resolve the upstream and downstream cost barriers associated with PHB production. Their greatest commercial potential is therefore likely to be realised when combined with complementary strategies such as copolymerisation, blending, and process optimisation.

Collectively, the modification strategies discussed in this section highlight the multifaceted effort required to overcome the inherent limitations of PHB. Copolymerisation improves material performance at the molecular level, blending enables property tailoring through complementary polymers, plasticisers enhance flexibility and processability, while fillers and nanocomposites provide opportunities for mechanical reinforcement. However, each strategy introduces additional considerations related to cost, processing complexity, scalability, or sustainability. Consequently, the successful commercialisation of PHB is unlikely to depend on any single modification approach, but rather on the development of integrated material systems capable of balancing performance requirements with economic feasibility.

Since all these modification approaches alter PHB performance, robust characterisation becomes essential to determine whether property improvements justify their additional complexity and cost.

### 3.5. Characterisation of Microbially Derived PHB

Successful PHB commercialisation depends not only on efficient production but also on the ability to consistently characterise polymer quality, composition, and performance. Variations in microbial strain selection, fermentation conditions, feedstock composition, extraction methods, and post-processing modifications can significantly influence key material attributes such as molecular weight, crystallinity, thermal stability, mechanical properties, and biodegradability [31]. Consequently, robust characterisation is essential for quality control, process optimisation, regulatory compliance, and product certification.

**Figure 6:**
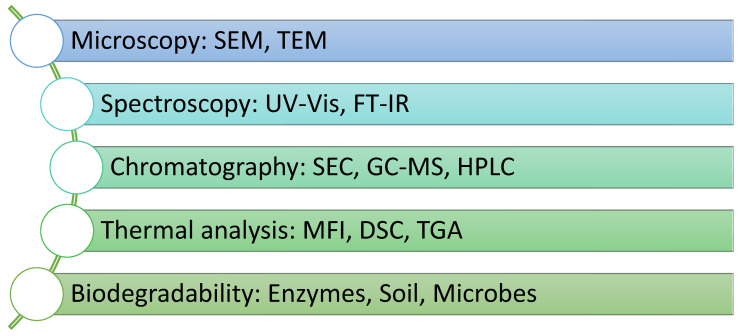
Summary of the principal analytical methods currently used for the detection, quantification, and characterisation of PHAs and PHB materials.

A wide range of analytical techniques have been developed for the screening of PHB-producing microorganisms and the characterisation of PHB materials. These methods can broadly be categorised into staining, spectroscopy, chromatography, thermal analysis, and microscopy techniques [159] illustrated in Figure 6.

While many of these techniques were originally developed for laboratory-scale investigations, they increasingly serve an important role in industrial PHB production by enabling process monitoring, product standardisation, and verification of material performance.

From a commercial perspective, reliable characterisation reduces uncertainty across the PHB value chain by ensuring that material specifications are consistently achieved and maintained. This becomes particularly important as PHB moves from laboratory-scale production towards larger-scale manufacturing, where reproducibility, quality assurance, and compliance with application-specific requirements become critical determinants of market adoption.

Having discussed the production, modification, and characterisation of PHB, the next critical aspect concerns its end-of-life behaviour and environmental performance. Since biodegradability is one of the principal advantages driving interest in PHB, understanding how the material degrades under different environmental conditions is essential for evaluating its sustainability and commercial potential.

### 3.6. Degradation Behaviour and Environmental Performance

One of the principal advantages driving interest in PHB is its ability to undergo complete biological degradation in a wide range of natural environments. Unlike conventional polyolefins such as polyethylene and polypropylene, which may persist in the environment for decades, PHB can be mineralised by naturally occurring microorganisms in soil, freshwater, marine environments, composting systems, and anaerobic digestion facilities [160]. End products include carbon dioxide, methane and new microbial biomass. This biodegradation capability has positioned PHB as one of the most environmentally attractive bioplastics currently available and is frequently cited as a key justification for its commercial development.

However, the commercial significance of biodegradability extends beyond environmental performance alone. For PHB to achieve widespread adoption, its degradation characteristics must translate into tangible market advantages that compensate for the higher production costs, processing challenges, and performance limitations discussed in previous sections. Consequently, understanding the relationship between polymer structure, degradation behaviour, and end-of-life management is critical when evaluating the overall commercial viability of PHB.

#### 3.6.1. Factors Influencing PHB Degradation

PHB degradation occurs primarily through enzymatic hydrolysis mediated by extracellular depolymerases secreted by bacteria and fungi [161,162]. These enzymes cleave ester linkages within the polymer backbone, generating oligomers and monomers that can subsequently be assimilated as carbon and energy sources by microorganisms [162]. The polymer-degrading microorganisms secrete extracellular enzymes and convert the PHB into molecular building blocks. A wide variety of extracellular depolymerase enzymes have been identified from a variety of microbes, and the mechanism is well understood [25]. Under aerobic conditions, degradation ultimately yields carbon dioxide and water, whereas anaerobic degradation produces methane and carbon dioxide.

The rate of degradation is strongly influenced by both environmental conditions and polymer characteristics. Factors such as temperature, moisture content, microbial population density, nutrient availability, pH, and oxygen availability all affect degradation kinetics [14,160,161,162,163]. From a material perspective, crystallinity remains one of the most important controlling parameters. Highly crystalline PHB structures are generally more resistant to enzymatic attack because the tightly packed polymer chains reduce enzyme accessibility [14,33]. Consequently, modifications that reduce crystallinity, such as copolymerisation, plasticisation, or blending, often improve both flexibility and biodegradation rates [164]. Standardised certifications reflect these dynamics; for instance, unmodified thin films regularly achieve greater than 90% carbon conversion within 180 days in laboratory marine trials (ASTM D6691) [165], whereas thick, highly crystalline rigid parts exhibit significantly extended environmental persistence.”

Several studies have reported that PHAs can achieve substantial degradation within weeks under industrial composting conditions [160,166,167], while degradation in marine environments occurs over considerably longer periods [162,163,168,169]. These observations highlight an important distinction between biodegradability and degradation rate; although PHB is biodegradable across diverse environments, the timescale of degradation remains highly dependent on environmental conditions. However, pure PHB possesses several inherent performance limitations, including poor melt strength and unfavourable physical–mechanical properties. Consequently, modifying PHB through innovative formulation approaches has become a critical requirement for developing viable, biodegradable materials tailored for diverse practical applications, as summarised in Figure 5. As detailed previously in Section 3.4, various strategies can be adopted to improve processability and mechanical performance; however, these structural modifications can severely alter and often impede the material’s subsequent biodegradation kinetics in natural environments.

#### 3.6.2. Environmental Advantage and Commercial Reality

The environmental performance of PHB represents one of its strongest competitive advantages relative to both conventional plastics and several commercially established bioplastics [164,170]. Unlike biobased but non-biodegradable polymers such as Bio-PET, PHB offers a genuine end-of-life solution through biological mineralisation. Furthermore, PHB degradation does not generate persistent microplastic residues and typically produces non-toxic degradation products, enhancing its suitability for environmentally sensitive applications [170,171].

Despite these advantages, biodegradability alone has not been sufficient to drive widespread market adoption. In practice, purchasing decisions are often governed by material cost, processability, and performance rather than end-of-life behaviour [164,172]. As discussed in the technoeconomic assessment, PHB production costs remain significantly higher than those of commodity plastics, while challenges associated with brittleness, thermal instability, and downstream recovery continue to constrain large-scale implementation.

This creates a fundamental commercial paradox. The environmental attribute that differentiates PHB most strongly from conventional plastics is realised primarily after product disposal, whereas the economic penalties associated with PHB are incurred throughout production and processing [172,173]. Consequently, the value proposition of PHB is strongest in applications where biodegradability is either legally mandated or provides a clear functional benefit, such as agricultural films, compostable packaging, biomedical devices, and marine-sensitive applications.

Therefore, while biodegradability remains a critical component of PHB’s sustainability profile, its contribution to commercial competitiveness depends on whether environmental regulations, carbon reduction targets, and circular economy policies can generate sufficient market value to offset the material’s higher production costs. The future success of PHB is therefore likely to depend not only on improvements in manufacturing economics but also on the continued evolution of regulatory frameworks that reward biodegradable and biobased materials.

### 3.7. Industrial Applications and Commercial Outlook

The diverse physicochemical properties of polyhydroxyalkanoates (PHAs), including biodegradability, biocompatibility, renewability, and tunable mechanical performance, have enabled their investigation across a broad range of industrial sectors. Depending on monomer composition and molecular architecture, PHAs can be tailored for applications ranging from packaging and agriculture to biomedical devices and specialty chemicals. These characteristics have positioned PHAs, particularly PHB and its copolymers, among the most promising candidates for replacing selected fossil-derived plastics within emerging circular bioeconomies.

However, despite decades of research and successful demonstration across numerous applications, large-scale market penetration remains limited. The commercial deployment of PHB is influenced not only by technical performance but also by production cost, processing requirements, regulatory drivers, and competition from both conventional plastics and alternative biopolymers. Consequently, the most commercially relevant applications are those in which the environmental or functional benefits of PHB justify its higher manufacturing costs.

#### 3.7.1. Packaging and Single Use

Packaging remains the largest potential market for PHB and other PHA-based materials [115,172]. PHB exhibits mechanical properties comparable to polypropylene while offering complete biological mineralisation across diverse terrestrial and aquatic environments, including soil, compost, freshwater, and marine systems. Rather than an absolute timeframe, this degradation is context-dependent, ranging from rapid assimilation within weeks under industrial composting configurations (55–60 °C) to extended windows spanning several months or years in ambient marine settings depending on the surface-to-volume ratio [14]. These characteristics make PHB attractive for disposable packaging, shopping bags, food-service items, agricultural mulch films, paper coatings, and other short-lifetime products where end-of-life management is a major concern [170,172].

Growing regulatory restrictions on single-use plastics have further strengthened interest in biodegradable alternatives [172]. Several countries have introduced taxes, bans, or extended producer responsibility schemes aimed at reducing plastic waste, creating market opportunities for biodegradable materials [164,172]. In such applications, PHB offers a clear environmental advantage over conventional polyolefins and partially biobased materials such as Bio-PET.

Nevertheless, packaging also represents the most price-sensitive plastics sector. Commodity polymers such as polyethylene and polypropylene remain substantially cheaper than PHB, often by a factor of three to five [103,173]. Consequently, PHB currently occupies niche markets where biodegradability commands a premium value rather than competing directly with commodity plastics on cost alone. This highlights a recurring theme throughout the PHB value chain: technical suitability does not automatically translate into commercial competitiveness.

#### 3.7.2. High-Value Biomedical Applications

Unlike packaging, biomedical applications are less sensitive to raw material costs and place greater emphasis on biocompatibility, safety, and functional performance.

An immense amount of clinical and material research continues to focus on applying PHAs such as PHB, PHBV, P4HB, PHBHHx, and PHO within the biomedical and tissue engineering sectors [154,156,157,174]. Over the past three decades, various laboratories and pre-clinical studies have utilised PHAs to prototype highly specialised medical devices. These include cartilage replacements, suture fasteners, orthopaedic pins, bone tissue scaffolds, nerve conduits, meniscus repair devices, adhesion barriers, heart valves, and cardiovascular patches [154,155,156,157,174].

The translational leap from lab bench to commercial reality was anchored by TephaFLEX^®^, the first FDA-cleared PHA-based absorbable suture [164]. Today, the industrial value chain spans from high-density microbial fermentation to fine chemicals and implantable medical devices. Following Becton, Dickinson and Company’s (BD) acquisition of Tepha, these proprietary Poly-4-hydroxybutyrate (P4HB) polymers have shown comparative potential to clinical standards and have been commercialised widely under major product lines like Phasix™ Mesh for soft tissue and hernia reconstruction and GalaFLEX^®^ bioresorbable scaffolds for plastic and reconstructive surgery.

From a cellular biology perspective, P(3HB) has been thoroughly proven to actively promote cell proliferation in tissue engineering models [46,175]. Empirical data demonstrate that P(3HB) at a concentration of 0.02 g.ml^−1^ significantly accelerates the proliferation of L929 plated cells when seeded at high density (1 × 10^5^ cells/well) while concurrently acting to inhibit programmed cell death [175]. Crucially, these polymer treatments accomplish this without disrupting the natural progression of the cell cycle, actively preventing cell necrosis and preserving the integrity and permeability of the cell membrane during the tissue-remodelling process.

#### 3.7.3. PHB-Based Targeted Drug Delivery Systems

Polyhydroxybutyrate is highly suited for targeted drug delivery due to its biocompatibility, biodegradability, and hydrophobic nature, which allows for highly efficient encapsulation of poorly water-soluble therapeutics (such as anticancer or antiviral agents) [154,176]. Unlike bulk-eroding polymers, PHB degrades via surface erosion, ensuring a predictable and controlled drug release rate [176,177]. Furthermore, its degradation byproduct, 3-hydroxybutyrate, is a natural human ketone body that is safely cleared by the body without toxicity [178].

A sophisticated, receptor-mediated delivery system leverages PhaP (phasin), a natural granule-associated protein with a strong hydrophobic affinity for the PHB surface [179]. By genetically engineering *E. coli* or *P. pastoris* to express cell-specific targeting ligands fused to PhaP, these proteins spontaneously self-assemble onto the PHB nanoparticles:

- Macrophage Targeting: PHB nanoparticles functionalized with mannosylated human α1-acid glycoprotein successfully target and enter macrophages.
- Tumour Targeting: PHB nanoparticles coated with human epidermal growth factor (hEGF) specifically bind to overexpressed receptors on hepatocellular carcinoma cells (BEL7402).

Using rhodamine B isothiocyanate (RBITC) as a fluorescent model drug, in vivo tracking has confirmed that these ligand–PhaP–PHB complexes are selectively endocytosed by tumour cells in xenograft mouse models, highlighting the system’s diagnostic and therapeutic efficacy potential [176,179].

Although biomedical applications represent a comparatively small market by volume, they currently provide one of the strongest commercial opportunities for PHAs because the high value of medical products can absorb the elevated production costs associated with microbial biopolymer manufacture.

#### 3.7.4. Emerging and Niche Applications

Beyond its traditional use in consumer packaging, PHB is carving out high-value niches in specialised sectors. Because these applications can utilise unpurified or crude PHB fractions, they offer excellent economic viability without requiring expensive downstream polymer purification.

(a) Controlled-Release Matrices and Agricultural Carriers

In both biomedicine and agriculture, PHB serves as an ideal material for smart, responsive delivery systems. It degrades steadily via predictable surface erosion, allowing it to act as a physical barrier that releases encapsulated payloads at a linear, optimal rate [3].

  ○ Biomedicine: PHB matrices allow the targeted, sustained release of therapeutic drugs directly at pathological sites, minimising systemic side effects [176].
  ○ Agriculture: Microbe-derived PHB matrices are used to encapsulate agrochemicals, pheromones, and fertilisers. These carriers release active ingredients into the soil over extended periods, drastically reducing environmental runoff and preventing the typical chemical wash-off caused by heavy rains [180].

(b) Speciality Coatings

PHB’s natural hydrophobicity and structural stability make it a highly effective biobased barrier coating. When applied to agricultural mulch films, paper-based food service packaging, or speciality textiles, thin PHB films significantly boost water resistance and gas-barrier properties [143,144,181]. Unlike synthetic petroleum-based coatings, PHB-coated materials remain fully compostable, degrading naturally into non-toxic soil nutrients at the end of their useful lifespans [160].

(c) Biofuel Precursors

Crude PHB extracted directly from wastewater-treatment sludge or industrial organic waste represents a promising, non-food-competitive feedstock for renewable energy [182]. Through acid-catalysed methyl esterification, the polymer is converted into R-3-hydroxybutyrate methyl ester (3HBME) [183]. This monomer exhibits a combustion heat of 20 kJ. g^−1^, which is promising but requires further optimisation to be comparable to ethanol (27 kJ. g^−1^) [182,183]. When blended into standard 97# gasoline at ratios between 5% and 20%, it acts as an effective oxygenated fuel additive rather than a direct substitute for ethanol, optimising viscosity, flash point, and octane performance without competing with human or animal food lifecycles [183].

**Figure 7:**
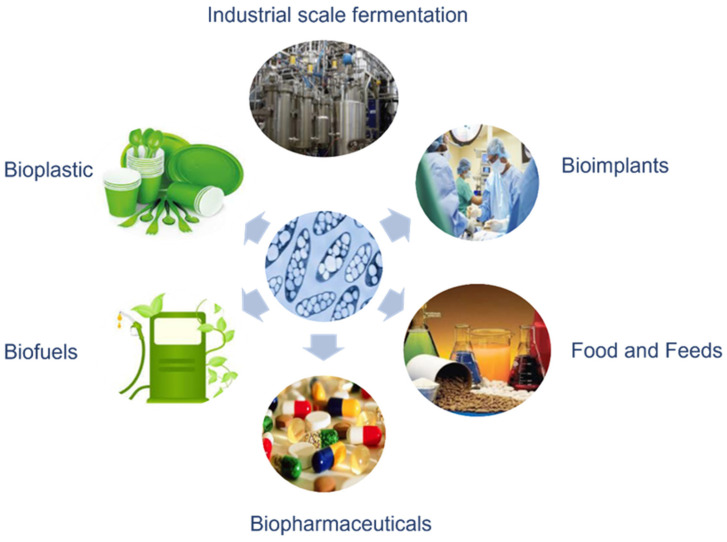
Summary of the principal industrial sectors targeted for or currently utilising PHB.

In Figure 7 below, the principal industrial sectors currently targeted for PHB commercialisation are summarised. While packaging represents the largest potential volume market, biomedical and pharmaceutical applications continue to offer the highest value-added opportunities due to the inherent biocompatibility and biodegradability of PHB.

While technically promising, most of these applications remain at laboratory or pilot scale. Commercial adoption is constrained by production economics, regulatory approval requirements, and competition from established materials with mature supply chains.

#### 3.7.5. Commercial Outlook for PHB

The commercial future of PHB will likely depend on the convergence of technological innovation, policy support, and market demand for sustainable materials. Advances in microbial engineering, utilisation of low-cost waste feedstocks, continuous fermentation strategies, and environmentally benign downstream processing have demonstrated significant potential to reduce production costs. However, current manufacturing costs remain substantially higher than those of commodity plastics and generally exceed those of competing bioplastics such as PLA.

Consequently, PHB is unlikely to replace conventional plastics across all market segments in the near term. Instead, commercialisation is expected to proceed through targeted applications where biodegradability, biocompatibility, or environmental performance provide a measurable economic advantage. The evolving PHB industry demonstrates that commercialisation strategies are increasingly centred on feedstock innovation, carbon utilisation, and waste valorisation rather than solely improving polymer properties. Representative examples of current PHB producers and technology platforms are summarised in Table 7.

Packaging for regulated markets, marine-degradable products, agricultural materials, and biomedical devices therefore represent the most realistic pathways for near-term growth.

Ultimately, the long-term success of PHB will depend on whether ongoing improvements in production efficiency and material performance can narrow the cost gap with established polymers while maintaining the sustainability benefits that distinguish PHB from competing materials. The challenge facing the industry is therefore no longer demonstrating that PHB works but rather achieving economically competitive production at commercial scale.

## 4. Conclusions and Future Perspectives

### 4.1. Concluding Remarks

PHB remains one of the most promising biobased polymers owing to its complete biodegradability, biocompatibility, renewable origin, and material properties comparable to several conventional thermoplastics. Advances in microbial strain development, feedstock diversification, polymer modification, and processing technologies have significantly expanded the potential applications of PHB across packaging, biomedical, agricultural, and speciality material sectors.

Despite these advances, large-scale commercialisation remains constrained by a combination of technical and economic challenges. High feedstock costs, oxygen transfer limitations during fermentation, contamination risks, downstream recovery costs, and the inherently narrow processing window of PHB continue to restrict competitiveness against commodity plastics and even more established bioplastics such as PLA. Although copolymerisation, blending, plasticisation, and composite strategies have improved material performance, these modifications often introduce additional processing complexity and cost.

### 4.2. Future Perspectives

Future research should focus on three interconnected priorities: (i) development of robust microbial platforms capable of utilising low-cost waste-derived feedstocks; (ii) implementation of environmentally benign and economically viable downstream recovery technologies; and (iii) design of application-specific PHB formulations that balance performance, processability, and cost. Advances in synthetic biology, metabolic engineering, and integrated biorefinery concepts are expected to play an important role in achieving these objectives.

The future success of PHB will ultimately depend not only on improvements in polymer properties but also on the ability to integrate feedstock sourcing, fermentation, recovery, and product manufacturing into economically sustainable value chains. As regulatory pressure against persistent plastics increases and circular bioeconomy initiatives continue to expand, PHB is well positioned to transition from a niche speciality biopolymer toward broader commercial adoption. However, achieving this transition will require continued innovation across the entire production-to-application pathway to overcome the cost and scalability barriers that currently limit market penetration.

## Figures and Tables

**Table 1:** General chemical structure of polyhydroxyalkanoates with various R-groups.

Number of Carbon Atoms	^+^ R Group	Polyhydroxyalkanoate Types	Abbreviation
* n = 1 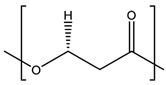	R = hydrogen (H)	Poly (hydroxy propionate)	P3HP
	R = methyl (CH_3_)	Poly (3-hydroxy butyrate)	P3HB
	R = ethyl (C_2_H_5_)	Poly (3-hydroxy valerate)	P3HV
	R = propyl (C_3_H_7_)	Poly (3-hydroxy hexanoate)	P3HH
	R = pentyl (C_5_H_11_)	Poly (3-hydroxy octanoate)	P3HO
	R = nonyl (C_10_H_10_)	Poly (3-hydroxy dodecanoate)	P3HD
**n = 2** 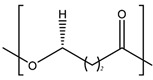	R = hydrogen (H)	Poly (4-hydroxy butyrate)	P4HB
	R = methyl (CH_3_)	Poly (4-hydroxy valerate)	P4HV
**n = 3** 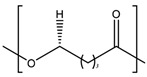	R = hydrogen (H)	Poly (5-hydroxy valerate)	P5HV
	R = methyl (CH_3_)	Poly (5-hydroxy hexanoate)	P5HH

^+^ The side-chain substituent “R” represents either a hydrogen atom or an alkyl group (e.g., methyl, ethyl, propyl, pentyl, or nonyl); * “n” denotes the number of methylene spacer groups in the polymer backbone (where n = 1 corresponds to 3-hydroxyalkanoates, n = 2 to 4-hydroxyalkanoates, and n = 3 to 5-hydroxyalkanoates).

**Table 2:** Comparison of the physical properties of PHB with various PHAs and with conventional fossil-based polymers.

Polymer	Young’s Modulus GPa	Tensile Strength MPa	Melting Temperature °C	Glass Transition Temperature °C	Elongation at Break %	Refs.
P(3HB)	3–4	40	173–180	5–9	3–10	[5,28,42,45]
P(4HB)	0.15	104	53–60	−50	1000	[46,47]
P(3HB-co-3HV) (3 mol% 3HV)	2.9	38	170	0–5	7	[48,49,50]
P(3HB-co-3HV) (25 mol% 3HV)	0.7	30	137	−4 to −5	120–800	[50,51,52]
P(3HB-co-4HB) (3 mol% 4HB)	2–3.2	28	166	2–3	45	[50,53]
P(3HB-co-4HB) (90 mol% 4HB)	0.1	65	50	−42	1080	[54,55,56]
P(3HHx-co-3HO)	<0.01	1–10	40–60	−30 to −40	100–300	[48,57,58]
P(3HB-co-3HA) (6 mol% 3HA)	0.5–1	20–30	133	−1 to −5	680	[59,60,61]
P(3HB-co-HP) (67 mol% HP)	<0.01	10–20	44	<0	100–400	[60]
Isotactic polypropylene	1.0–1.8	30–40	160–166	−10–0	100–600	[32,60,62]
HDPE	0.4–1.2	17.9–43	112–137	−80	500–1000	[63,64]
LDPE	0.15–0.3	10–25	88–130	−125 to −100	150–600	[65,66]
Nylon-6,6	2.8–4	70–90	255–265	50–90	20–70	[67,68]
Polyethylene-terethalate	2.8–3.1	55–75	262	~70	50–300	[69,70]

Key: HDPE—high-density polyethylene; LDPE—low-density polyethylene.

**Table 3:** Microbial systems for PHB production: performance, substrate flexibility, and processing trade-offs.

Category	Microorganism	Carbon Source	PHB Yield (% *w*/*w*)	Extraction Method	Key Insight	Refs.
High-yield industrial strains	*Cupriavidus necator*	Vegetable oil	~93	Solvent (cyclohexanone)	Benchmark organism; high yield but dependent on controlled conditions	[79]
	*Bacillus thuringiensis*	Sugarcane juice	60–72	Solvent (chloroform)	Good yields on agro-based substrates; solvent recovery remains a challenge	[80]
Substrate-flexible systems	*Halomonas boliviensis*	Starch hydrolysate|Quinoa stalks	~56	Solvent (chloroform)	Can utilise low-cost substrates; potential for non-sterile processing	[81]
	*Methylobacterium* sp.	Methanol	~38	Solvent (chloroform)	Enables use of C1 feedstocks; introduces toxicity and process control challenges	[85]
	*Serratia* sp.	Xylose	~37	Solvent (methanol)	Compatible with lignocellulosic streams; require pretreatment	[87]
Waste-derived/low-cost feedstock systems	*Azotobacter vinelandii*	Wheat bran	~43	Chemical digestion	Low-cost substrate but polymer purity may be compromised	[86]
	*Burkholderia sacchari*	Glucose/Sucrose	40–60	Soxhlet extraction	Industrial potential but extraction method is energy-intensive	[83,84]
Alternative/engineered systems	*Escherichia coli* BL21	Glucose	~80–87	Enzymatic	Enables controlled synthesis; lower yield and higher cost	[92]
Low yield/niche systems	*Spirulina platensis*	CO_2_	~30	Solvent (methanol)	Sustainable but not yet industrially viable	[93]

**Table 4:** Sustainable feedstocks for PHB production: advantages, limitations, and commercial relevance.

Feedstock Category	Examples	Advantages	Key Limitations	Commercial Relevance	Refs.
Refined sugars (1st Gen)	Glucose, sucrose, fructose	High yields; stable fermentation	High Cost; food competition	Benchmark substrate for high-purity PHAs but costly	[105]
Plant & waste oils	Vegetable oil, waste cooking oil (WCO)	High PHB accumulation.	Mass transfer & purification challenges	Strong near-term potential but requires specialised reactor design.	[106]
Lignocellulosic biomass (2nd gen)	Wheat straw, sugarcane bagasse, corn stover	Renewable non-food carbon source	Pretreatment & inhibitor formation	Sustainable but technically intensive	[102,107]
Industrial by-products	Crude glycerol, molasses, cheese whey	Low-cost waste valorisation	Impurities & metabolic constraints	Attractive for integrated biorefineries	[11]
Volatile fatty acids (VFAs)	Acetate, propionate, butyrate	Efficient metabolic precursors	Toxicity at high concentrations	Promising for waste-based PHB production	[108]
Wastewater streams	Municipal sewage, palm oil mill effluent	Simultaneous remediation & production	Variable composition & consistency	Suitable for mixed-culture systems.	[109]
C1 Substrates & Gases (3rd/4th Gen)	Methanol, CO_2_, CH_4_, syngas (CO/H_2_)	Enables carbon capture	Gas–liquid transfer and safety limitations	Emerging long-term platform	[110]

**Table 5:** Comparative economic position, estimated market price, and key commercial limitations of PHB relative to competing bioplastics and conventional plastics.

Material Category	Polymer Type	Estimated Market Price (USD. kg^−1^)	Approximate Commercial Position (2024–2026)	Key Commercial Limitation	Refs.
PHA Bioplastics	PHB	$3.5–11	Emerging (<5% of bioplastics market)	High production & downstream recovery costs	[103,121,122]
Bioplastics	PLA (Polylactic Acid)	$1.8–3	Dominant bioplastic (~32–37%)	Poor thermal resistance and brittleness	[123,124]
	Starch Blends	$1.6–4.5	Moderate market presence	Moisture sensitivity; reduced mechanical strength	[6]
	PBAT	$1.5–3.5	Growing biodegradable polymer segment	Fossil-based origin and relatively high cost	[125]
	PBS	$2.5–5.0	Niche market (<2%)	Limited production scale and relatively high cost	[126]
	Bio-PET	$1.4–2.0	Major drop-in bioplastic (~24–26%)	Non-biodegradable despite partial biobased origin	[127]
Fossil-based Plastics	LDPE	0.9–1.5	Commodity polymer	Non-biodegradable and fossil-derived	[128,129]
	Polypropylene (PP)	$1.0–2.0	Major commodity plastic	Environmental persistence and fossil dependence	[62,103]
	Polystyrene (PS)	$1.3–1.7	Mature commodity polymer	Brittleness and poor environmental degradability	[130]
	PET (Virgin)	$1.0–1.5	Major packaging polymer	Fossil-derived and persistent plastic waste generation	[103,131]

**Table 6:** Common biodegradable polymers used in PHB/PHBV blend systems and their primary functional role.

Polymer	Origin & Synthesis	Glass Transition (T_g_) & Melting (T_m_) Temperatures (°C)	Mechanical Profile	Role in PHB/PHBV Blends	Refs.
PLA (Polylactic Acid)	100% biobased (ring-opening polymerisation of lactide)	T_g_~55–60 T_m_~150–180	High strength (~60 MPa); High modulus; Low elongation at break (<10%); Highly brittle	Improves rigidity and tensile strength	[9,123,124,134]
PBAT (Polybutylene Adipate Terephthalate)	Aliphatic-aromatic copolyester (petroleum-based)	T_g_~−30 T_m_~110–120	Low tensile strength (~20 MPa); High elongation at break (>500%); High tear toughness	Improves ductility, elongation and toughness	[13,136,137]
PBS (Polybutylene Succinate)	Aliphatic polyester (bio-succinic acid or petrochemical)	T_g_~−32 T_m_~114–115	Moderate tensile strength (~34 MPa); Good impact resistance; Thermal stability	Improves processability and impact resistance	[3,13]
TPS (Thermoplastic Starch)	Destructured natural starch (plasticised with glycerol/water)	Dominated by plasticiser content (T_g_ < 0)	Highly hydrophilic; Lacks dimensional stability	Reduces material cost and improves sustainability profile	[3,138]

**Table 7:** Current commercial PHB production industries.

Company	Location	PHB Technology Platform	Production Scale (2025 Updates)	Primary Applications & Status
Newlight Technologies, Inc.	Huntington Beach, California, USA	AirCarbon^®^ (PHB derived from greenhouse gases/methane)	Commercial Scale (Expanding)	High-end consumer goods; Packaging applications
Biomer GmbH	Krailling, Bavaria, Germany	PHB formulations: Biomer^®^ P226/P209; Biomer^®^ P263/P300 AND P304	Pilot scale	Injection moulding & technical parts; Packaging applications; Biomedical devices
TianAn Biopolymer Co., Ltd.	Ningbo, Zhejiang, China	Pure PHB & PHBV blends	~2000 t/a	Resins; Blends; Packaging applications
PHB Industrial S.A	Sao Paulo, Sao Paulo State, Brazil	Biocycle^®^ (PHB from sugarcane molasses)	Pilot/Small Industrial	Eco-packaging & cosmetics.
Bluepha Co., Ltd.	Beijing, Beinjing Municipality, China	Bluepha^®^ PHA (Mainly homopolymer PHB variants)	25,000 t/a (Total site capacity)	Mass-market packaging
Genecis Bioindustries, Inc.	Toronto, Ontario, Canada	PHB from organic food waste	Pilot to Demo Scale	Circular packaging & resins
Full Cycle Bioplastics LLC	San Jose, Carlifonia, USA	PHB from cellulosic waste/organic waste	Pilot Scale	Licensing technology
CO2BioClean GmbH	Dusseldorf, North Rhine-Westphalia, Germany	PHB produced from industrial CO_2_ emissions	Pilot Scale	Biomedical & textiles.
KANEKA Corp.	Osaka, Osaka Prefecture, Japan	Green planet™ (PHBH product produced from plant oils)	20,000 t/a	Packaging & films
Becton, Dickinson & Co Inc.	Franklin Lakes, New Jersey, USA	BD Phasix™ Mesh and GalaFLEX^®^ Scaffold Portfolio produced from P4HB	Commercial Scale	Medical applications

## Data Availability

No new data were created or analyzed in this study. Data sharing is not applicable to this article.
